# A Pancreatic Solid Pseudo-Papillary Tumor Detected After Abdominal Injury

**DOI:** 10.4021/gr534e

**Published:** 2013-05-03

**Authors:** Atsushi Ishii, Kazuko Yoshimura, Hiroshi Ideguchi, Shinichi Hirose

**Affiliations:** a Department of Pediatrics, School of Medicine, Fukuoka University, Japan

**Keywords:** Abdominal injury, Solid pseudo-papillary tumor of the pancreas, Magnetic resonance imaging, Neuron-specific enolase

## Abstract

Solid pseudo-papillary tumor (SPT) of the pancreas is a relatively benign tumor that is more frequently reported in females. Most patients usually present with abdominal pain or mass. We experienced the girl who identified SPT with the injury. We diagnosed SPT in a previously healthy 14-year-old Asian girl after abdominal injury. She experienced upper abdominal pain and vomiting after being hit by a basketball. Blood examination revealed a high serum amylase level. Abdominal radiography indicated abnormal bowel gases. Contrast-enhanced computed tomography revealed a smooth, peripheral and unilocular mass approximately 55 mm in diameter in the pancreatic tail. Based on these observations, acute pancreatitis complicated by a pancreatic mass was initially diagnosed. Therapy for acute pancreatitis was instituted, while we simultaneously investigated the mass. Levels of tumor markers were not profoundly elevated in serum. Dynamic contrast-enhanced magnetic resonance imaging (MRI) revealed moderate and gradual increase in contrast-enhanced imaging, consistent with findings of SPT of the pancreas. We thus elected surgical resection for her. Pathological examination of the surgical specimen confirmed our diagnosis of SPT. SPT of the pancreas should be considered as a differential diagnosis of acute abdomen disorders, especially in instances after minor abdominal injuries in young women, and diagnoses must be confirmed with MRIs.

## Introduction

Solid pseudo-papillary tumor (SPT) of the pancreas is a rare tumor that is predominantly reported in young women [1-4]. Findings of SPT indicate a low-grade malignant potential and presentation of a relatively large mass [1, 3, 4]. The incidence of malignant transformation is approximately 15% [1, 4]. Most patients usually present with abdominal pain or mass. However, 9% patients are asymptomatic and tumors are accidentally detected after abdominal trauma [3, 5]. SPT usually appears as an encapsulated mass composed of a mixture of cystic, solid and hemorrhagic components [3, 4, 6, 7]. SPT is pathologically characterised by a solid and cystic portion with papillary arrangement of tumor cells [1, 5, 8, 9]. SPT is usually diagnosed by various imaging studies. Magnetic resonance image (MRI) is particularly helpful in diagnosing SPT. MRI provides superior tissue contrast and consistent concurrence with histopathological findings.

## Case Report

**Figure 1:**
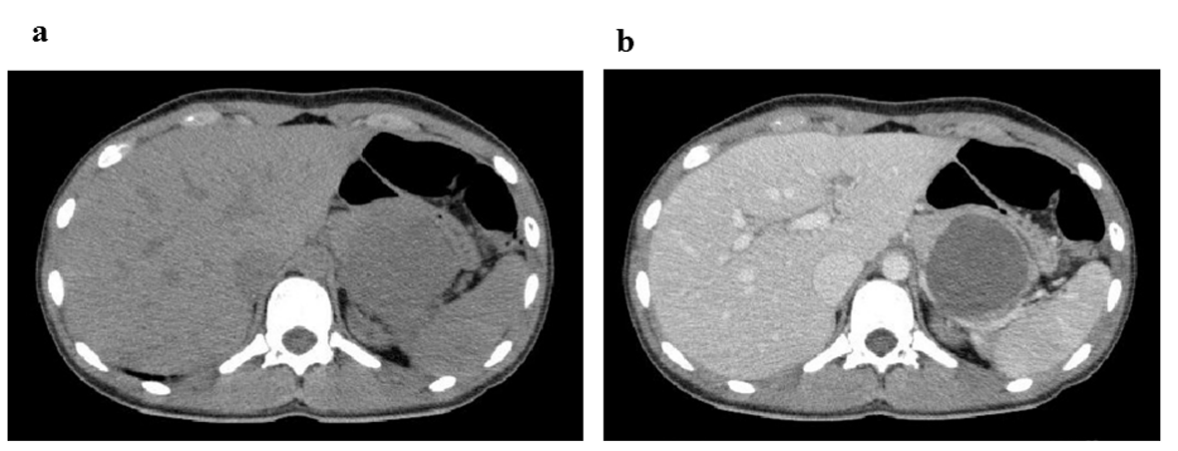
CT images at 4 h after abdominal injury. (a) Plain CT reveals a cystic mass measuring 55 mm in the pancreatic body and tail. (b) Contrast-enhanced CT indicates a heterogeneous internal density and smooth rim enhancement of the mass.

**Figure 2:**
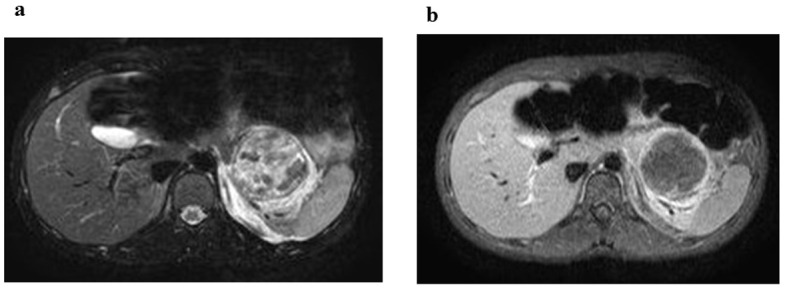
MRI reveals a mass approximate 50 mm in size in the pancreas. (a) T2-weighted MRI reveals a mass, measuring 50 mm approximately, in the pancreatic tail with associated heterogeneous high intensity that indicates the presence of an encapsulated solid and cystic mass with areas of hemorrhagic degeneration. (b) Dynamic contrast-enhanced MRI demonstrates contrast enhancement in the early arterial phase; the contrast enhancement gradually increases in the late phases.

The patient was a 14-year-old Asian girl with no abnormal perinatal, medical or surgical history. She presented to the emergency medical centre for outpatients with abdominal pain and vomiting 3 h after being struck in the abdomen by a basketball. She had a slight fever but her other vital signs were stable. Her abdominal examination was revealed spontaneous pain and tenderness in the left upper quadrant. Plain abdominal radiography revealed abdominal intestinal gas and blood examination indicated moderately elevated serum amylase level. Hospitalisation was deemed necessary and she was transferred to our university hospital. She was hospitalised 4 h after injury. Blood tests were repeated (Table 1) and they indicated an increase in white blood cell count and serum amylase level. However, other blood test results were normal. Free air and the colon cutoff sign were absent on abdominal radiography. However, abnormal bowel gas, which indicates paralytic ileus was observed in the abdomen X-rays. Computed tomography (CT) of the abdomen and pelvis was performed to rule out traumatic intra-abdominal injury (Fig. 1a, b). CT revealed a cystic mass measuring 55 mm at the pancreatic body and tail (Fig. 1a) with associated heterogeneous internal density and smooth rim enhancement (Fig. 1b). We diagnosed the patient with acute pancreatitis complicated by pancreatic pseudocyst, hematoma or tumor. Intravenous administration of urinastatin and gabexate mesilate was initiated and continued till 11 days after the onset of illness, after which oral administration was feasible. MRI was performed at 4 days after the onset of illness (Fig. 2a, b). T2-weighted MRI revealed a mass measuring approximately 50 mm at the pancreatic tail with associated heterogeneous high intensity that indicated an encapsulated solid and cystic mass with areas of hemorrhagic degeneration (Fig. 2a). The mass showed minimal contrast enhancement on dynamic contrast-enhanced MRI in the early arterial phase; the contrast enhancement gradually increased in the late phases (Fig. 2b). The mass was diagnosed as SPT of the pancreas based on these MRI findings. Profound increases in CEA, CA 19-9, AFP and neuron-specific enolase (NSE) levels were not observed on investigation (Table 1). SPT of the pancreas has low-grade malignant potential; therefore, we performed total-body CT to confirm the absence of metastases. The patient underwent central pancreatectomy at 28 days after the onset of illness. Final pathology revealed SPT that was 41 mm in greatest dimension. Tissue sections showed extensive hemorrhage, necrosis and granulation tissue. Solid portions of the tumor revealed sheets of uniform round or oval nuclei and a vacuolated cytoplasm. The tissue in the cystic region of the tumor assumed a pseudo-papillary appearance projecting into the space filled with hemorrhagic and necrotic debris. The patient recovered from surgery and was discharged with no reports of hepatic dysfunction.

**Table 1:** Laboratory Data on Admission and Tumor Markers

Item	Value	Unit
WBC	11.2	10^3^/µL
Neutrophil	85.8	%
Lymphocyte	11.1	%
RBC	484	10^4^/µL
Hb	13.4	g/dL
Plt	23.5	10^4^/µL
Protein	8.0	g/dL
Albumin	4.8	g/dL
UN	12	mg/dL
Cr	0.6	mg/dL
Na^+^	141	mmol/L
K^+^	4.4	mmol/L
Cl^−^	104	mmol/L
Ca^2+^	9.6	mg/dL
Total bilirubin	0.3	mg/dL
AST	17	IU/L
ALT	17	IU/L
ALP	411	IU/L
γ-GTP	14	IU/L
CK	315	IU/L
Amylase	287	U/L
Glucose	130	mg/dL
CRP	0.0	mg/dL
PT	11.1	sec
APTT	24.9	sec
Fibrinogen	292	mg/dL
D-dimer	< 0.5	mg/mL
CAE	0.8	ng/mL
CA19-9	11	U/mL
AFP	1.7	ng/mL
NSE	22	ng/mL

## Discussion

SPT of the pancreas was first described as a papillary cystic tumor by Frantz in 1959. At present, based on the World Health Organization classification (1996), the appropriate terminology for this tumor is SPT of the pancreas. SPT predominantly affects young females in their second or third decades of life and can present in childhood [1, 2]. As for clinical course, SPT has a low-grade malignant potential and the prognosis is almost excellent. Post-operative 5-year survival rates as high as 95% have been reported [3, 4]. Gross pathological findings of SPT reveal round and well-encapsulated tumors composed of solid, cystic and papillary portions accompanied by hemorrhage and necrosis [3, 4, 6, 7]. Microscopic assays demonstrate a high cellular area composed of small epithelioid cells that form sheets or papillary structures around fibro-vascular cores [1, 8, 9].

SPT is difficult to diagnose based on clinical findings. The most common presentation is abdominal pain or a palpable mass. Approximately 15% patients are asymptomatic and SPT is often detected accidentally in such cases [3]. Laboratory data do not indicate specific abnormalities for SPT or even elevated levels of specific tumor markers such as CEA, CA19-9 and CA125. However, elevated NSE levels have been reported [9, 10].

In particular, MRI aids in pre-operative diagnosis of SPT. MRI findings of SPT reflect histopathological features. T2-weighted MRI demonstrates a well circumscribed tumor with moderate high intensity representing the predominantly solid portion and low intensity representing the predominantly cystic portion. T1-weighted MRI reveals high intensity associated with hemorrhage within the lesion. The enhancement pattern demonstrates a gradual enhancement of contrast dye within the tumor, which shows early arterial enhancement.

In conclusion, MRI should be performed to rule out SPT in cases of young women presenting with a pancreatic mass lesion on CT early after abdominal injuries. SPT must be considered as differential diagnosis in such instances in particular.
